# Multiple Cross Displacement Amplification Coupled with Lateral Flow Biosensor (MCDA-LFB) for rapid detection of *Legionella pneumophila*

**DOI:** 10.1186/s12866-021-02363-3

**Published:** 2022-01-08

**Authors:** Luxi Jiang, Rumeng Gu, Xiaomeng Li, Meijun Song, Xiaojun Huang, Deguang Mu

**Affiliations:** 1 Department of Respiratory Medicine, Zhejiang Provincial People’s Hospital, People’s Hospital of Hangzhou Medical College, Hangzhou, Zhejiang People’s Republic of China; 2 Graduate School of Clinical Medicine, Bengbu Medical College, Bengbu, China; 3 Department of Respiratory and Critical Care Medicine, Shanghai Fourth People’s Hospital Affiliated to Tongji University, 1279 Sanmen Road, Shanghai, 200080 People’s Republic of China; 4 Department of respiratory and critical care medicine, Xi’an Daxing Hospital, Xi’an, China

**Keywords:** *L. pneumophila*, Multiple cross displacement amplification, Lateral flow biosensor, MCDA-LFB, Limit of detection

## Abstract

**Background:**

*Legionella pneumophila* is an opportunistic waterborne pathogen of significant public health problems, which can cause serious human respiratory diseases (Legionnaires’ disease). Multiple cross displacement amplification (MCDA), a isothermal nucleic acid amplification technique, has been applied in the rapid detection of several bacterial agents. In this report, we developed a MCDA coupled with Nanoparticles-based Lateral Flow Biosensor (MCDA-LFB) for the rapid detection of *L. pneumophila*.

**Results:**

A set of 10 primers based on the *L. pneumophila* specific *mip* gene to specifically identify 10 different target sequence regions of *L. pneumophila* was designed. The optimal time and temperature for amplification are 57 min and 65 °C. The limit of detection (LoD) is 10 fg in pure cultures of *L. pneumophila*. No cross-reaction was obtained and the specificity of MCDA-LFB assay was 100%. The whole process of the assay, including 20 min of DNA preparation, 35 min of *L. pneumophila*-MCDA reaction, and 2 min of sensor strip reaction, took a total of 57 min (less than 1 h). Among 88 specimens for clinical evaluation, 5 (5.68%) samples were *L. pneumophila*-positive by MCDA-LFB and traditional culture method, while 4(4.55%) samples were *L. pneumophila*-positive by PCR method targeting *mip* gene. Compared with culture method, the diagnostic accuracy of MCDA-LFB method was higher.

**Conclusions:**

In summary, the *L. pneumophila*-MCDA-LFB method we successfully developed is a simple, fast, reliable and sensitive diagnostic tool, which can be widely used in basic and clinical laboratories.

**Supplementary Information:**

The online version contains supplementary material available at 10.1186/s12866-021-02363-3.

## Background

*Legionella* species are opportunistic pathogens that are widely found in moist soil, freshwater environment and compost materials [1]. It is the causative agent of Legionnaires’ disease (LD), which has two clinical manifestations in humans: Legionnaires’ disease and Pontiac fever [2]. Legionnaires’ disease is characterized by severe lung infection symptoms, including severe pneumonia with a high fatality rate. So far, there are more than 65 different species of *Legionella*. Among them, *Legionella pneumophila* (*L. pneumophila*) accounts for more than 90% of the Legionnaires’ disease [3]. Humans are mainly infected by inhaling contaminated water aerosols in “public” and “private” places [4]. At present, due to changes in the global climate and rainfall, coupled with the increase in the proportion of susceptible people (the elderly, immunodeficiency, etc.), the incidence of Legionnaires’ disease is increasing year by year globally [5]. A report from the European Center for Disease Prevention and Control (ECDC) showed that 9328 cases of Legionnaires’ disease were reported in 2017, an increase of 30% over 2016 (https://www.ecdc.europa.eu/en/search?s=Legionnaires%27+Disease+in+Europe%2C+Annual+Epidemiological+Report+for+2017). In 2015, a report showed that there were 1.3 deaths from Legionnaires’ disease per 100,000 individuals in Europe [6]. Due to the symbiotic interaction between *Legionella* and free-living amoeba (FLA) [7], and the biofilm-related *Legionella* has strong resistance to disinfectants and biocides [8].it is difficult for us to control *L. pneumophila* in the polluted environment resources. However, environmental monitoring for *L. pneumophila* is essential in assessing risks and determining treatment strategies. In addition, for high-risk groups, rapid detection of *L. pneumophila* infection is critical to clinical prognosis. Therefore, it is urgent to establish a rapid and accurate diagnostic method to guide the treatment of early infection of *L. pneumophila.*

Diagnostic methods, including traditional bacterial culture methods, serological testing, urine antigen detection and nucleic acid amplification techniques, have been developed and used to detect Legionnaires’ disease. The traditional culture method is considered to be the golden standard for detecting *L. pneumophila* and is widely used in clinical and environmental detection. However, it has many limitations, such as laborious, time-consuming (sometimes up to 2 weeks), poor sensitivity [9]. and can’t detect viable but non-culturable cells [10]. Serological testing has low sensitivity. The urinary antigen detection can only detect *L. pneumophila* serogroup 1 (Sg 1), not sensitive to other species [2, 11]. To date, several nucleic acid test methods have been developed to identify *L. pneumophila*, including conventional PCR, multiplex PCR, quantitative PCR, and real-time PCR [12–14]. These methods have high specificity and sensitivity, and the detection is rapid and reproducible. However, these PCR-based methods require expensive equipment, complicated process and professional personnel that are not suitable in most clinical institutions. Therefore, a simple timely, labor-saving, and efficient detection method to detect *L. pneumophila* is urgently needed.

Multiple cross displacement amplification (MCDA), a novel isothermal nucleic acid amplification technique, has been applied in detecting many bacterial agents [15–18]. MCDA assay utilizes a polymerase with strand displace activity to amplify the target. It requires 10 primers to achieve the sequence-based amplification at a fixed temperature (61–68 °C), which binds to 10 regions of target sequences. Therefore, only a water bath or simple heater that maintained a fixed temperature was sufficient. Moreover, the nanoparticle-based lateral flow biosensor (LFB) was used to analyze MCDA products. In the LFB assay, the sample is directly applied onto the sample pad. Driven by capillary force, sample solution would automatically flow from sample pad to absorbent pad. And target analyte causes aggregation of reporter molecules on the test zone, leading to appearance of a visible line on the test zone. Its results are less subjective and does not require any sophisticated instruments [19, 20]. Then, the combination of MCDA and LFB (MCDA-LFB) is simple, rapid, highly sensitive and specific.

In this report, based on the specific *mip* gene, the MCDA-LFB assay for the rapid detection of *L. pneumophila* was successfully developed and verified. Then, the sensitivity and specificity of the MCDA-LFB assay were assessed, and 88 samples (sputum, alveolar lavage fluid and water samples) were tested using the MCDA-LFB assay. In addition, we compared the test results from MCDA-LFB method with the traditional culture and PCR methods.

## Results

### Demonstration and detection of *L. pneumophila*-MCDA products

In order to determine the effectiveness of the *L. pneumophila* MCDA primers (Table 1), the MCDA assay with DNA from pure cultures of *L. pneumophila* were carried out at 65 °C for 35 mins. The results were shown in Fig. 2. The DNA from *L. pneumophila* (ATCC 33152) was significantly amplified, while no amplification was observed from the DNA of *Klebsiella pneumoniae* (ATCC 12657), *Listeria monocytogenes* (ATCC 49593) and the double distilled water (DW) control. Therefore, *L. pneumophila*-MCDA primer set targeting *mip* gene was a good candidate for the development of MCDA-LFB method for *L. pneumophila* detection.

**Table 1:** The Primers Used in the Current Report

Primers Name^**a**^	Sequences and modifications^**b**^	Length^**c**^	Gene
F1	5′-GAAATGGTGTTAAACCCGG-3′	19 nt	*mip*
F2	5′-AAGTTTCATTTGGGCCAAT-3′	19 nt	
CP1	5′-GGTACTGTCAAAAACGGTACCACGGATACAGTCACTGTCGA-3′	41mer	
CP2	5′-TGGATGGACAGAAGCTTTGCGGCCATATGCAAGACCTGA-3′	39mer	
C1*	5′-Biotin-GGTACTGTCAAAAACGGTACCA-3’	22mer	
C2	5′-TGGATGGACAGAAGCTTTGC-3’	20 nt	
D1*	5′-FITC-TCAATCAGACGACCAGT-3’	17 nt	
D2	5′-TGCCAGCTGGATCAACTT-3’	18 nt	
R1	5′-GTTGCTGGCTTACCAGT-3’	17 nt	
R2	5′-GTTCCAGGTTTCACAAGTT-3’	19 nt	

^a^C1∗, 5′-labeled with biotin when used in MCDA-LFB assay; D1∗, 5′-labeled with FITC when used in MCDA-LFB assay

^b^FITC, fluorescein isothiocyanate

^c^nt, nucleotide; mer, monomeric

### Optimal amplification temperature for the *L. pneumophila*-MCDA-LFB assay

The MCDA reaction was performed at eight different temperatures (61–68 °C, with 1 °C interval) to determine the optimal amplification temperature. The DNA concentration of each reaction was 10 pg/mL, and the results of all reactions were monitored with the real-time turbidity method. Faster amplification was observed at an experimental temperature of 65 °C (Fig. 3). Therefore, 65 °C was selected as the optimal amplification temperature and used for the remaining MCDA reactions in this report.

### Specificity of MCDA-LFB for *L. pneumophila* detection

The genomic DNA of the bacteria listed in Table 2 were used to analyze the specificity of *L. pneumophila*-MCDA-LFB method. As shown in Fig. 4, only the DNA of *L. pneumophila* obtained positive results (Fig. 4, B1), and two crimson lines (TL and CL) appeared simultaneously on the biosensor. The DNA of other non-*L. pneumophila* strains did not show positive result (Fig. 4, B2-B20), only a crimson line (CL) appeared on the sensor. Meanwhile, visual detection reagent (VDR) and gel electrophoresis were used to detect the specificity of MCDA-LFB assay (Fig. 4A and C), and the results were completely consistent with LFB.

**Table 2:** Bacterial strains used in this study

Bacteria	Strain no./source^**a**^	No. of strains	MCDA-LFB result^**b**^
*Legionella pneumophila*	ATCC 33152	1	P
	Isolated strains (ZJ)	23	P
*Vibrio parahaemolyticus*	Isolated strains (ZJ)	1	N
*Listeria monocytogenes*	ATCC 49593	1	N
*Enterococcus faecalis*	Isolated strains (ZJ)	1	N
*Streptococcus bovis*	Isolated strains (ZJ)	1	N
*Klebsiella pneumoniae*	ATCC 12657	1	N
*Shigella boydii*	Isolated strains (ZJ)	1	N
*Yersinia enterocolitica*	Isolated strains (ZJ)	1	N
*Streptococcus suis*	Isolated strains (ZJ)	1	N
*Streptococcus pneumoniae*	ATCC 27336	1	N
*Bordetella parapertussis*	Isolated strains (ZJ)	1	N
*Acinetobacter baumannii*	Isolated strains (ZJ)	1	N
*Staphylococcus epidermidis*	Isolated strains (ZJ)	1	N
*Staphylococcus saprophyticus*	Isolated strains (ZJ)	1	N
*Mycoplasma*	ATCC 15377	1	N
*Bacillus proteus*	Isolated strains (ZJ)	1	N
*Bacillus cereus*	Isolated strains (ZJ)	1	N

^a^*ATCC* American T ype Culture Collection; (ZJ), Zhejiang provincial people’s Hospital

^b^*P* positive, *N* negative. Only *L. pneumophila* strains could be detected by the MCDA-LFB technique, indicating the extremely high selectivity of the method

### Sensitivity of MCDA-LFB for *L. pneumophila* detection

Serial dilutions of the *L. pneumophila* DNA templates were used to confirm the limit of detection (LoD) of *L. pneumophila*-MCDA-LFB assay. As shown in Fig. 5, the MCDA-LFB method was able to detect as low as 10 fg genomic DNA in each reaction. Two crimson bands (TL and CL) on the biosensor indicated the positive result of the *mip* gene (Fig. 5B), and the result obtained by the biosensor was completely consistent with the VDR analysis (Fig. 5A).

### Optimized the reaction time of MCDA-LFB for *L. pneumophila* detection

In this report, we determined the optimal reaction time for the *L. pneumophila*-MCDA assay during the amplification stage. Different amplification time was compared at the optimal amplification temperature (65 °C), ranging from 25 min to 55 min, with an interval of 10 min. The target DNA at the LoD level (10 fg per reaction) could be detected when the m-MCDA reaction only lasted for 35 min (Fig. 6). Thus, 35 min was selected as the optimal isothermal amplification time and used for the rest MCDA experiment. In short, the whole process, including DNA template preparation (20 min), *L. pneumophila*-MCDA reaction (35 min), and LFB results (2 min), took 57 min (less than 1 h) in total.

### Evaluation of the MCDA-LFB assay using clinical samples

A total of 88 samples were used to further verify the feasibility of the *L. pneumophila*-MCDA-LFB method. Informed consent has been obtained from all patients in the study. We used MCDA-LFB assay, conventional PCR method and culture method to detect these 88 samples. The results were summarized in Table 3. Among 88 samples, 4 (4.55%) samples were *L. pneumophila*- positive by traditional PCR method, and the other 84 samples were negative. The MCDA-LFB assay in this report detected 5 (5.68%) positive samples, which completely covered the 4 positive samples detected by PCR. This MCDA-LFB result was completely consistent with conventional culture method. Both of the two methods confirmed the same 5 positive samples. The above data showed that the MCDA-LFB assay developed in this report is a valuable detection tool for *L. pneumophila*, and has better detection ability than PCR method.

**Table 3:** Comparison of PCR, culture-biotechnical, and MCDA-LFB assays for the detection of *L. pneumophila* in sputum samples

Detection methods	Sputum samples (***n*** = 88)	
	Positive	Negative
MCDA-LFB	5	83
Culture	5	83
PCR	4	84

## Discussion

**Fig. 1:**
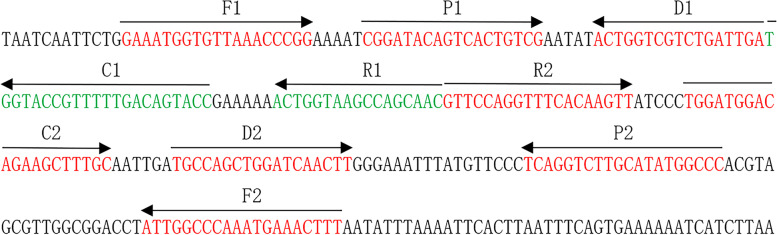
Positions and sequences of the primers on the *mip* gene. Positions and sequences of the *mip* gene used to design multiple cross displacement amplification primers. The nucleotide sequences of the sense strand of mip gene are listed. Right arrows and left arrows indicate sense and complementary sequences that are used

*L. pneumophila* is the causative agent of Legionnaires’ disease. It is an important public health problem that causes a large number of morbidity and mortality worldwide. Rapid detection of *L. pneumophila* is extremely important for the early treatment of Legionnaires’ disease. In the current study, we successfully established the MCDA-LFB assay targeting *mip* gene for detecting *L. pneumophila* strains. This assay has the advantages of simple operation, rapid results, high sensitivity and specificity, and is suitable for both laboratory and clinical. The *mip* gene, which encodes the peptidylprolyl *cis/trans* isomerase, is one of the first genes related to *L. pneumophila* replication in eukaryotic cells [21]. We designed 10 MCDA primers (Fig. 1) specifically identified 10 special regions of *mip* gene, thus ensuring a high specificity for *L. pneumophila* detection. Moreover, in order to confirm the specificity of the MCDA-LFB method, we examined the genomic DNA extracted from 40 strains (Table 2). The results showed that all *L. pneumophila* isolates were positive, and all non-*L. pneumophila* isolates were negative (Fig. 4). The above data indicated that the identification of *L. pneumophila* by MCDA-LFB method targeting the *mip* gene has 100% specificity.

**Fig. 2:**
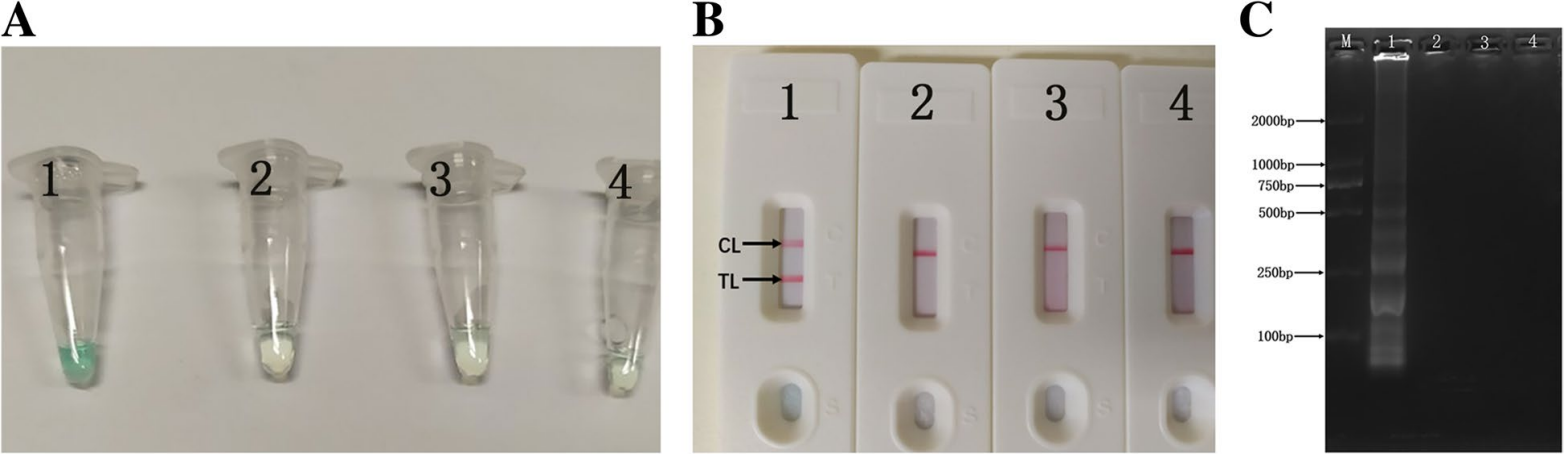
Demonstration and detection of MCDA products. **A** Color change of *mip*-MCDA tubes; **B** LFB used to visual detection of *mip*-MCDA products; **C** Agarose gel electrophoresis of mip-MCDA products. Tube A1/Biosensor B1/Lane C1, positive amplification of *L. pneumophila* strain; Tube A2/Biosensor B2/Lane C2, negative amplification of *Klebsiella pneumoniae* strain; Tube A3/Biosensor B3/Lane C3, negative amplification of *Listeria monocytogenes* strain; Tube A4/Biosensor B4/Lane C4, and blank control of DW

**Fig. 3:**
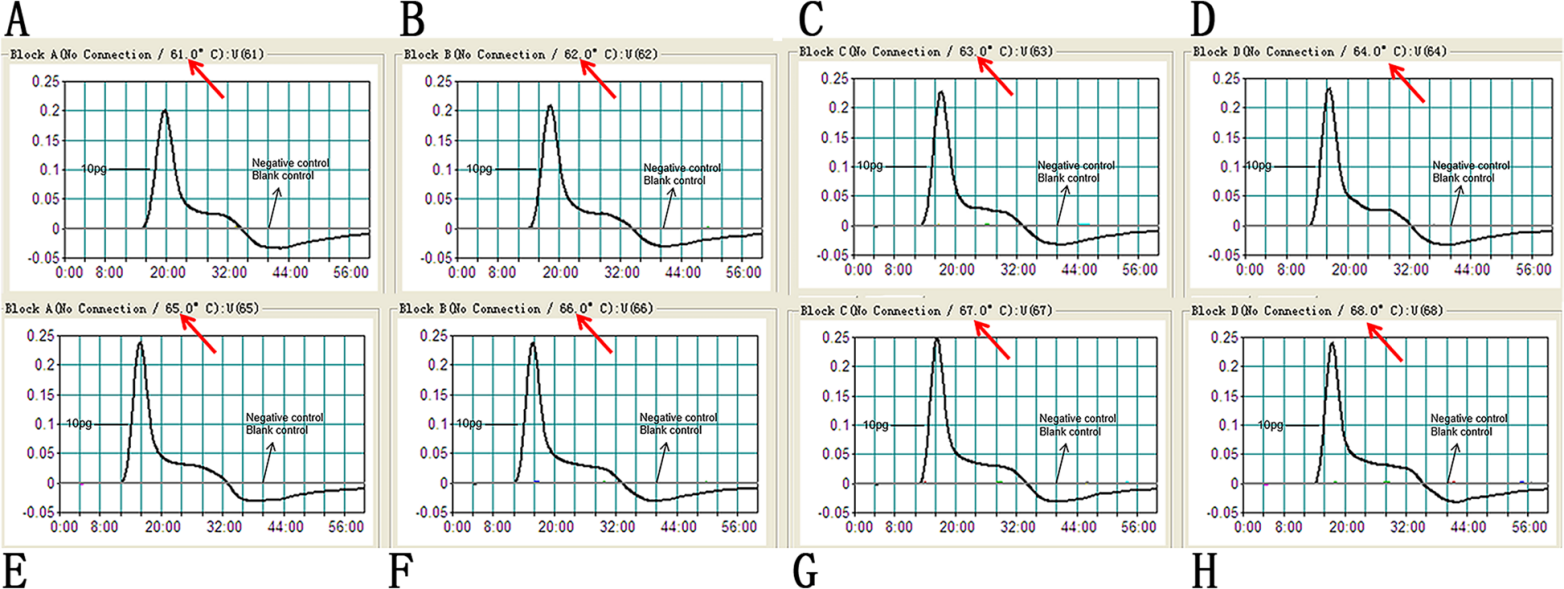
Optimal amplification temperature for MCDA assay. *L. pneumophila*-MCDA reactions were monitored by real-time measurement of turbidity. The corresponding curves were shown in the picture. A turbidity of > 0.1 indicated positive amplification of MCDA assay. Eight kinetic curves (**A–H**) were generated from 61 to 68 °C, with *L. pneumophila* 10 pg genomic DNA per reaction

**Fig. 4:**
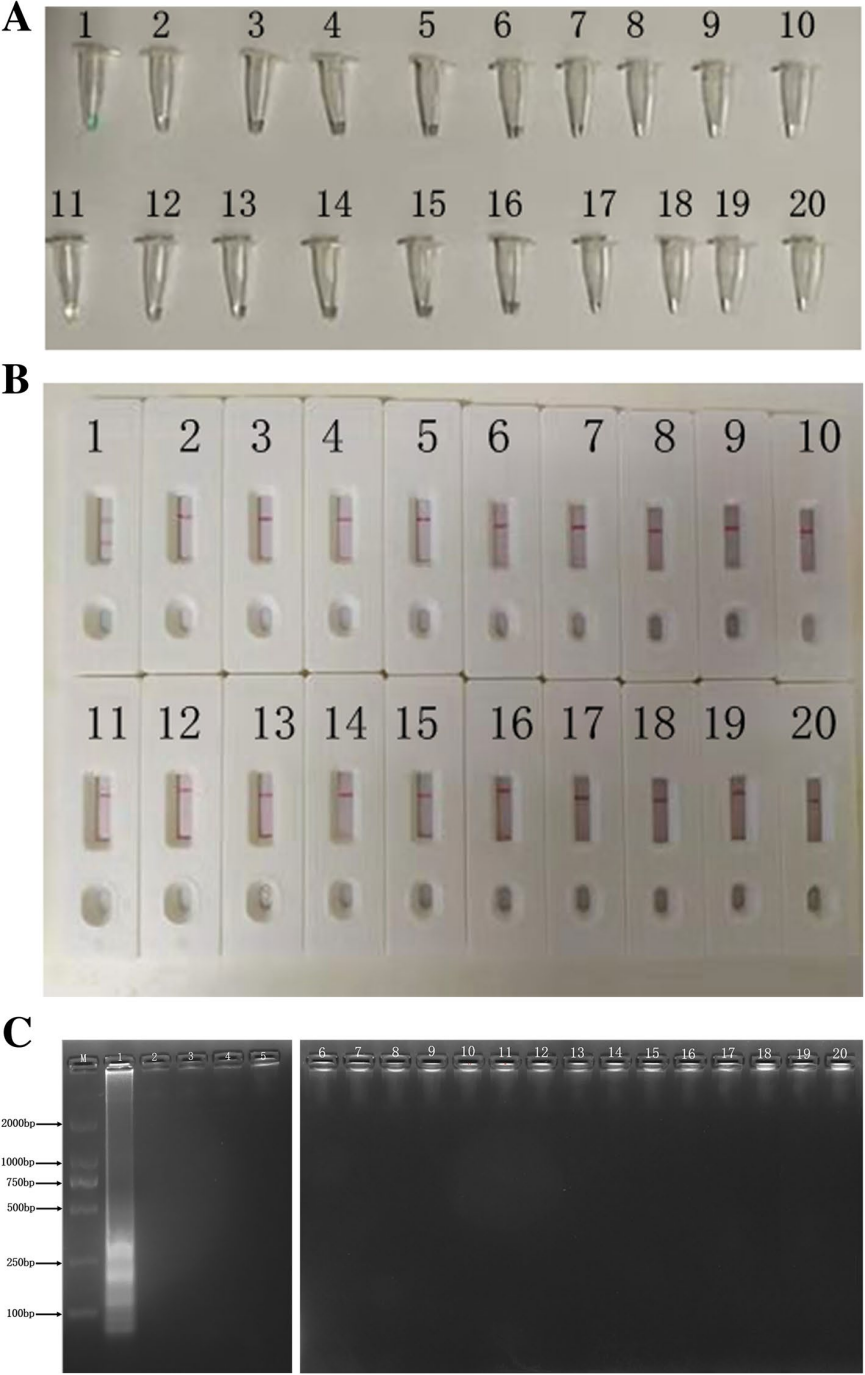
Specificity of the *L. pneumophila*-MCDA assay using different strains. The MCDA reactions were performed using different genomic DNA templates and were monitored by (**A**) VDR analysis, (**B**) lateral flow biosensor and (**C**) gel electrophoresis. Tube A1/Biosensor B1/ Lane C1, *L. pneumophila* (ATCC 33152); Tube A2-A19, Biosensor B2-B19 and Lane C2–19, represent 16 different non-*L. pneumophila* strains (details shown in Table 2); Tube A20, Biosensor B20 and Lane C20 represent blank control (DW). Supplementary note: Due to the number of lanes for one-time electrophoresis in our laboratory was less than 21, we performed two gel electrophoresis experiments

**Fig. 5:**
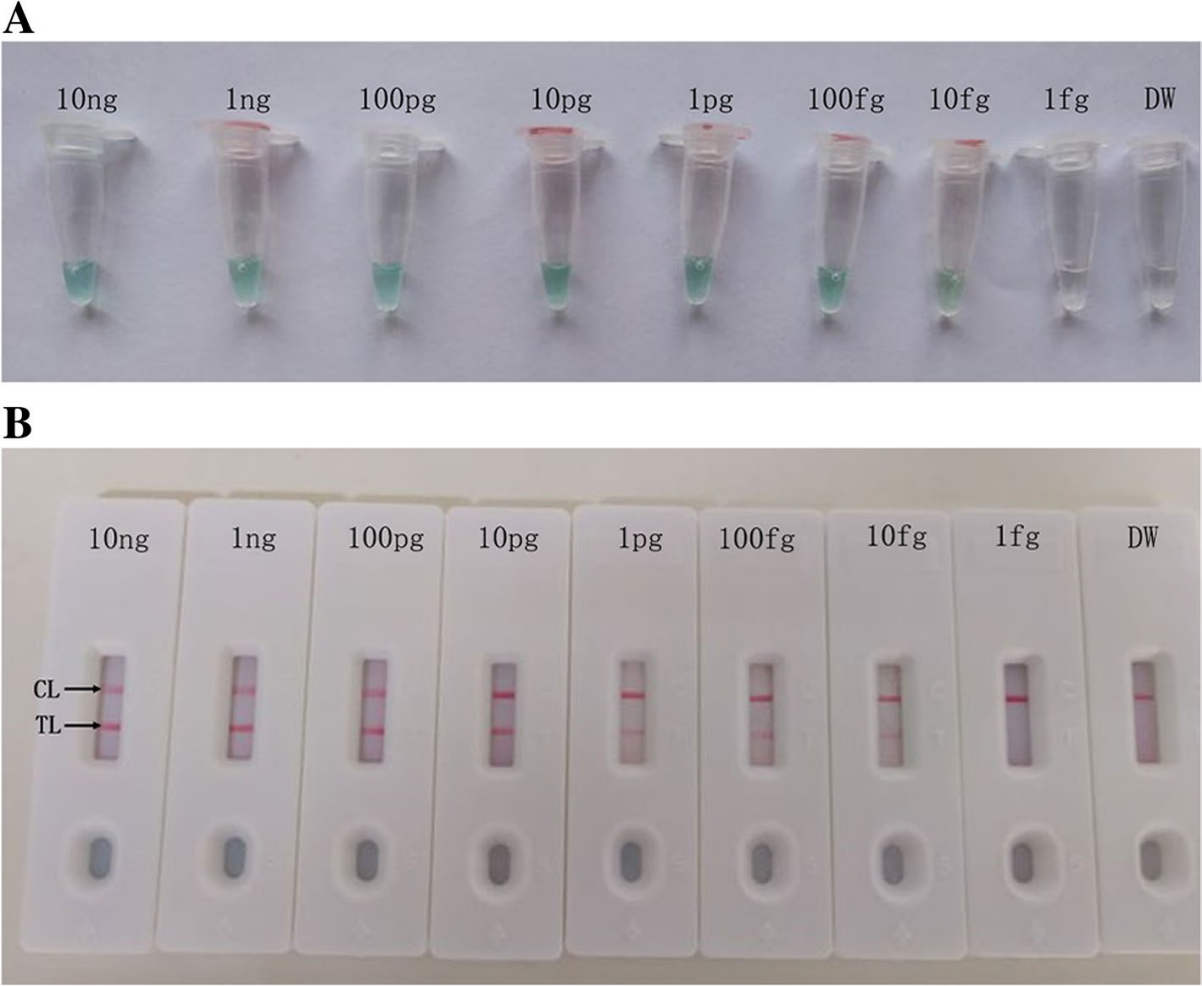
Sensitivity of the *L. pneumophila*-MCDA assay. Two detection techniques including (**A**) VDR analysis and (**B**) lateral flow biosensor were applied for MCDA products. Serial dilutions (10 ng/μL, 1 ng/μL, 100 pg/μL, 10 pg/μL, 1 pg/μL, 100 fg/μL, 10 fg/μL, 1 fg/μL) of *L. pneumophila* ATCC 33152 genomic DNA were used for sensitivity analysis. Tubes (**A**)/ Biosensor (**B**) 1–8 represented different concentrations of DNA, 10 ng/μL, 1 ng/μL, 100 pg/μL, 10 pg/μL, 1 pg/μL, 100 fg/μL, 10 fg/μL, 1 fg/μL, respectively; 9 represents a negative control (DW). The LoD of *L. pneumophila*-MCDA assay for *mip* gene detection was 10 fg per reaction

**Fig. 6:**
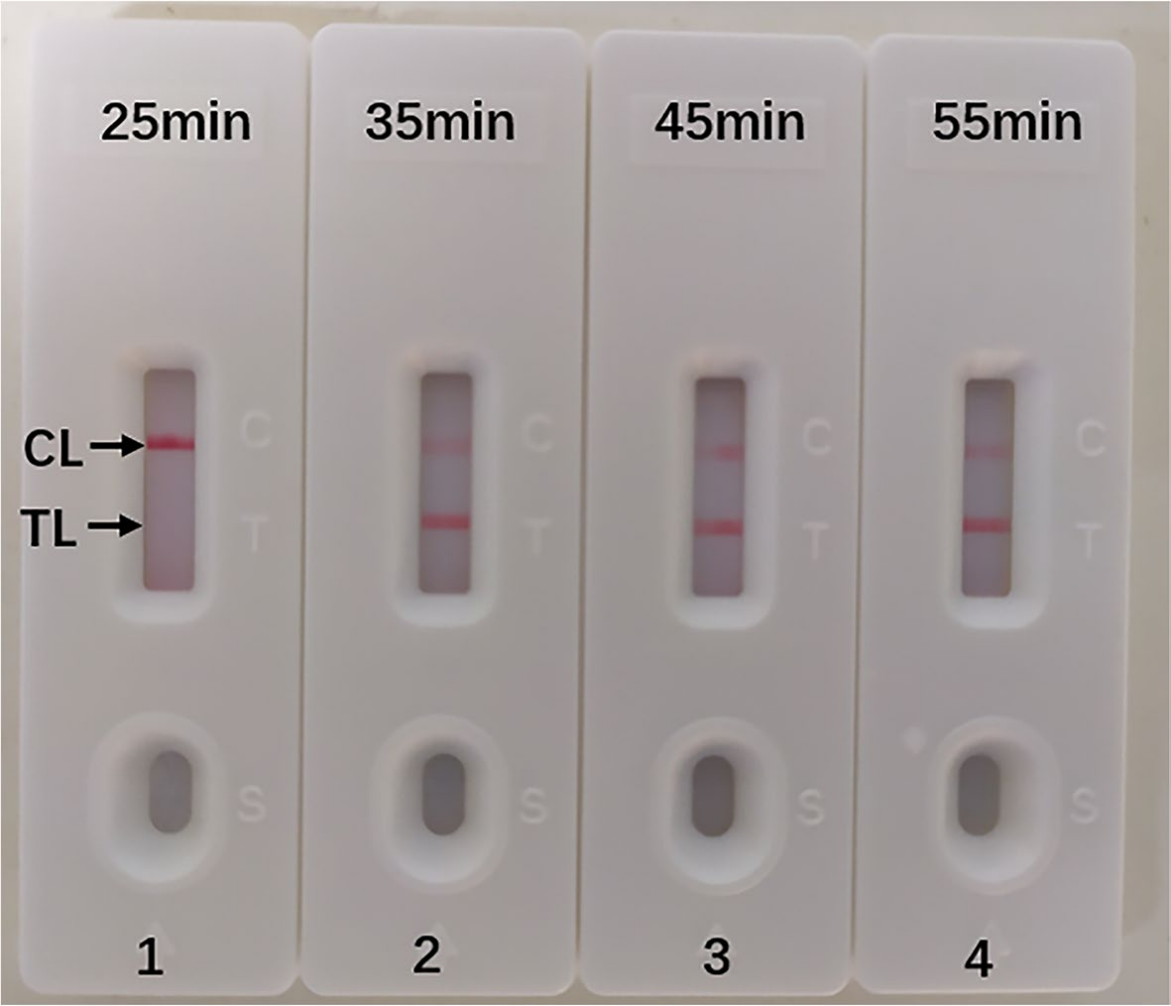
Optimized reaction time for MCDA assay. Four different reaction times (A, 25 min; B, 35 min; C, 45 min; D, 55 min) were tested and compared at optimal amplification temperature (65 °C). Biosensors 1, 2, 3 and 4 represent DNA template levels of 10 fg/μL (LoD level). The best sensitivity was seen when the amplification lasted for 35 min

In this assay, we used LFB to detect MCDA products (Figs. 2B, 3B, 4B, 5B and 6). Compared with the gel electrophoresis (Figs. 2C and 4C), real-time turbidity (Fig. 3) and VDR (Figs. 2A, 4A and 5A) used in this experiment, LFB has the advantages of simple operation, low error rate, and can also be used in field, clinical and laboratory. Especially in analyzing MCDA products, the LFB method did not require complicated equipment, special reagents and additional procedures. Furthermore, the cost of LFB is affordable, only $2 USD per test. And it only takes 2–3 h to produce about 1000 LFBs in batches. On average, the preparation time of each LFB is very short, which is very suitable for practical application. Therefore, compared with other detection techniques, LFB can display the amplification results of MCDA reaction more quickly, simply and intuitively.

In the MCDA-LFB method for the detection of *L. pneumophila*, we found that the method has high sensitivity. The limit of detection (LoD) is 10 fg *L. pneumophila* genomic DNA extracted from pure cultures. Meanwhile, we used VDR to further confirm the sensitivity of *L. pneumophila*, and LFB is as sensitive as VDR (Fig. 5). Compared to PCR-based methods, MCDA reaction only requires simple heating equipment, such as laboratory water bath equipment, dry heater or portable battery-powered heater, to provide a constant optimal amplification temperature of 65 °C. Therefore, the MCDA-LFB method can be used for clinical and environmental monitoring of *L. pneumophila* because of its simplicity, rapidity and high sensitivity.

The MCDA-LFB developed in this report can quickly detect the DNA of *L. pneumophila*, which is a time-saving and convenient molecular detection technology. The whole detection process took only 57 min (less than 1 h), including 20 min of DNA preparation, 35 min of *L. pneumophila*-MCDA reaction, and 2 min of LFB results reporting. Then, in order to further confirm the practical feasibility of the *L. pneumophila*-MCDA-LFB method. We detected 88 specimens collected from clinical patients and institute for communicable disease control and prevention by MCDA-LFB method, PCR method and traditional culture method. MCDA-LFB method and traditional culture method have the same test results (5 positive samples), while PCR analysis only detected 4 positive samples (Table 3). This data indicated that compared with PCR technology, MCDA-LFB showed higher sensitivity to target pathogens. The lower detection rate of PCR method may be due to the fact that the copy numbers of *L. pneumophila* gene templates in that sample were lower than the detection limit of traditional PCR method.

## Conclusion

In summary, we successfully built a simple, fast, and nearly instrument-free MCDA-LFB method for the detection of *L. pneumophila*. We designed a set of primers based on the *mip* gene to ensure high specificity for the detection of *L. pneumophila*, and all isolated strains of *L. pneumophila* were successfully detected. The method also showed a high level of sensitivity, with the LoD of 10 fg genomic DNA per reaction in a pure culture. In addition, we also used sputum, alveolar lavage fluid and water samples to confirm the practical feasibility of MCDA-LFB technology. Besides, the biosensor was used to analyze amplification products, which was fast, easy to operate, simple and objective. Therefore, the *L. pneumophila*-MCDA-LFB method we successfully developed is a simple, fast, reliable, and sensitive diagnostic tool, which can be widely used for the identification of *L. pneumophila* in basic and clinical laboratories.

## Materials and methods

### Reagents and instruments

Absorbent pads, nitrocellulose (NC) membranes, sample pads, conjugate pads and membrane backing materials were purchased from the Jie Yi Biotechnology Co., Ltd. (Shanghai, China). Isothermal amplification kits and visual detection reagent (VDR) were purchased from BeiJing-HaiTaiZhengYuan Technology Co., Ltd. (Beijing, China). Biotinylated bovine serum albumin (biotin-BSA) and rabbit anti-fluorescein antibody (anti-FITC Ab) were purchased from the Abcam Co., Ltd. (Shanghai, China). Dye (Crimson red) streptavidin coated polymer nanoparticles (129 nm, 10 mg mL^− 1^, 100 mM borate, pH 8.5 with 0.1% BSA, 0.05% Tween 20, and 10 mM EDTA) were purchased from Bangs Laboratories, INC. (Indiana, USA). Genomic template extraction kits (QIAamp DNA mini kits; Qiagen, Hilden, Germany) was purchased from Qiagen Co., Ltd. (Beijing, China).

### Bacterial strains and genomic template preparation

A total of 40 bacterial strains were used in this assay, including 24 strains of *L. pneumophila* and 16 strains of non-*L. pneumophila* (Table 2 and additional Table 1). *L. pneumophila* (ATCC 33152) was used as a reference strain to optimize MCDA assay. The original DNA template extraction concentration of ATCC 33152 was 200.9 ng/μL. The specific DNA extraction steps are as follows: sputum and alveolar lavage fluid specimens were pre-treated with proteinase K, ASL buffer, and buffer AL, and incubated for 15 min at 70 °C. DNA extraction was achieved using QIAamp DNA mini kits (Qiagen, Hilden, Germany). Filter the water sample (500 ml) with filter paper. After the water was filtered, cut the filter paper and rub the bacteria on the filter paper on the BCYE petri dish, and the DNA was extracted according to QIAamp DNA Kit. Then, the extracted genomic DNA was quantified by ultraviolet spectrophotometer (Nano drop one, Thermo, Beijing, China). DNA templates of *L. pneumophila* strain 33,152 were serially 10-fold diluted (10 ng/μL, 1 ng/μL, 100 pg/μL, 10 pg/μL, 1 pg/μL, 100 fg/μL, 10 fg/μL, 1 fg/μL) and 1 μL of each dilution was added into the reactions for sensitivity analysis of *L. pneumophila*-MCDA-LFB detection. And the DNA extraction concentration of samples used in this experiment ranged from 5 ng/μL to 53 ng/μL.

### Primer design

Based on the *mip* ge*ne* sequence of *L. pneumophila*, we designed 10 MCDA primers by PrimerExplorer V4 (Eiken Chemical, Japan) and primer software PRIME, RPREMIER 5.0, including two replacement primers (F1 and F2), six amplification primers (C1, C2, D1, D2, R1 and R2) and two cross primers (CP1 and CP2). To detect MCDA products by LFB, the 5′ ends of the C1 and D1 primers were labeled with biotin and FITC, respectively. Details of the positions, sequences and modifications of the primers are shown in Fig. 1 and Table 1. All primers were synthesized by TsingKe Biotech Co., Ltd. (Beijing, China).

### Preparation of gold nanoparticle-based dipstick biosensor

LFB is prepared according to previous reports [22, 23]. Briefly, the biosensor is mainly composed of an immersion pad, a backing pad, NC membrane, an absorbent pad, and a conjugate pad. SA-PNPs (dye) were gathered in the conjugate pad. Biotin-BSA and anti-FITC were affixed at the control line (CL) and test line (TL), respectively. And each line was separated by 5 mm. As a result, Crimson red lines were easily visible on the biosensor within 2 min.

### The standard MCDA assay

According to previous research [15, 24], *L. pneumophila*-MCDA reactions were performed in a 25-μl amplification mixtures. Each reaction mainly contained displacement primers F1 and F2 (0.1 μM each), amplification primers C1 *, C2, R1, R2, D1* and D2 (0.2 μM each), and cross primers CP1 and CP2 (0.4 μM each), DNA template (1 μl), 10 U *Bst* DNA polymerase (1.25 μl) and 12.5 μl 2 × reaction mixture (Loopamp DNA amplification kit).

There are four monitoring methods for *L. pneumophila*-MCDA amplicons in this assay, including real-time turbidity (LA-320C), visual detection reagent (VDR), gel electrophoresis and LFB detection. In VDR, when detecting amplification products, the color will change from colorless to light green, and it will remain colorless when detecting the negative control and blank control. The real-time turbidity meter LA-320 performs real-time analysis by recording the optical density (OD) of the MCDA reaction. MCDA products were analyzed by 3% agarose gel electrophoresis, the specific ladder of multiple bands should be seen for positive amplifications, but not in the negative and blank controls. By LFB, two visible crimson red lines (TL; CL) could be observed in positive reactions, and only the control lines were visual in negative and blank controls.

We then evaluated the optimal temperature of *L. pneumophila*-MCDA primers, ranging from 61 to 68 °C (with an interval of 1 °C). In addition, *Klebsiella pneumoniae* (ATCC 12657) and *Listeria monocytogenes* (ATCC 49593) were used as negative controls (NC), DW was used as a blank control (BC).

### Specificity of the *L. pneumophila*-MCDA-LFB assay

The DNA templates of 24 *L. pneumophila* strains and 16 non-*L. pneumophila* strains (Table 2) were used to evaluate the specificity of *L. pneumophila*-MCDA-LFB assay. All experiments were repeated for three times, and all MCDA results were shown with LFB.

### Sensitivity of *L. pneumophila*-MCDA-LFB assay

The sensitivity of MCDA-LFB method was evaluated using DNA template serial dilutions as described above. The limit of detection (LoD) was defined as the minimum dilution gradients that show a positive result. Ultimately, the MCDA-LFB detection results were compared with VDR.

### Optimization the reaction time of the *L. pneumophila*-MCDA-LFB assay

We evaluated the optimal reaction time of MCDA assay from 25 min to 55 min, with an interval of 10 min. And then we used LFB to detect all MCDA products. In addition, all reactions were repeated three times.

### Examination of the feasibility of MCDA-LFB assay in clinical samples

In order to test the feasibility of MCDA-LFB assay, we used traditional bacterial culture method, conventional PCR detection and MCDA-LFB method to test 88 specimens suspected of *L. pneumophila* infection collected from Zhejiang Provincial people’s Hospital and Shenyang Institute for Communicable Disease Control and Prevention (additional Table 2). the *Legionella*-positive samples come from different patients, the collection time is also different, there is no correlation between them. This study was approved by the Ethics Committee of the Zhejiang provincial people’s Hospital, and conducted according to the medical research regulations of the Ministry of Health, China. The collected samples were subjected to both acid and heat treatment. Then, the treated samples were inoculated on Buffered Charcoal Yeast Extract (BCYE) agar supplemented with l-cysteine and the plates were incubated in candle jar (3–5% CO2) at 37 °C in a humidified atmosphere and examined for 4–14 days for the presence of *Legionella* spp. colonies. Suspicious colonies with characteristics of *Legionella* were subcultured on BCYE agar with and without L-cysteine ​​and unselective media for verification. Isolates which grew on BCYE agar with L. cysteine, but not on the other media, were considered supposed *Legionella*. Subsequently the identification of *Legionella* spp. were done by biochemical tests [25], If biochemical tests, including gelatin liquefaction, amylase hydrolysis and hippuric acid tests are positive, nitrate reduction and urease tests are negative, it is considered positive for *Legionella*. Besides, *L. pneumophila* strains can also be identified by Gram staining and serum agglutination test. Simultaneously, Genomic DNA was extracted from sputum, alveolar lavage fluid and water samples by QIAamp DNA Kit, which is used as a template for MCDA-LFB and PCR detection. PCR was employed using *L. pneumophila* specific primers targeting *mip* gene [21], and the primer sequences were as follows : forward primer, 5′-GCAATGTCAACAGCAA −3′; reverse primer, 5′-CATAGCGTCTTGCATG-3′. Amplification was carried out in following conditions: an initial denaturation at 95 °C for 4 min, followed by 30 cycles at 94 °C for 30 s, 55 °C for 30 s, 72 °C for 30 s, and a final extension at 72 °C for 5 min [26]. Finally, we compared the results of MCDA-LFB method, culture and PCR methods.

## Supplementary Information

**Additional file 1.**

## Data Availability

The datasets supporting our findings are included in the article.

## Abbreviations

MCDA: Multiple cross displacement amplification
LFB: Lateral flow biosensor
TL: Test line
CL: Control line
FITC: Fluorescein isothiocyanate
ATCC: The Global Bioresource Center
DW: Distilled water
VDR: Visual detection reagent
NC: Nitrocellulose
DNA: Deoxyribo Nucleic Acid
WHO: World Health Organization
ECDC: European Center for Disease Prevention and Control

## References

[1] Newton HJ, Ang DK, van Driel IR, Hartland EL (2010). Molecular pathogenesis of infections caused by Legionella pneumophila. Clin Microbiol Rev.

[2] Fields BS, Benson RF, Besser RE (2002). Legionella and Legionnaires' disease: 25 years of investigation. Clin Microbiol Rev.

[3] Lesnik R, Brettar I, Höfle MG (2016). Legionella species diversity and dynamics from surface reservoir to tap water: from cold adaptation to thermophily. ISME J.

[4] Blatt SP, Parkinson MD, Pace E, Hoffman P, Dolan D, Lauderdale P (1993). Nosocomial Legionnaires' disease: aspiration as a primary mode of disease acquisition. Am J Med.

[5] Walker JT (2018). The influence of climate change on waterborne disease and Legionella: a review. Perspect Public Health.

[6] Beauté J. Legionnaires' disease in Europe, 2011 to 2015. Euro Surveill. 2017;22(27):171116-1. 10.2807/1560-7917.ES.2017.22.27.30566 PMC5508329 28703097

[7] Valciņa O, Pūle D, Mališevs A, Trofimova J, Makarova S, Konvisers G, et al. Co-Occurrence of Free-Living Amoeba and Legionella in Drinking Water Supply Systems. Medicina (Kaunas, Lithuania). 2019;55(8):492. 10.3390/medicina55080492 PMC6723719 31443316

[8] Abu Khweek A, Amer AO (2018). Factors mediating environmental biofilm formation by Legionella pneumophila. Front Cell Infect Microbiol.

[9] Boulanger CA, Edelstein PH (1995). Precision and accuracy of recovery of Legionella pneumophila from seeded tap water by filtration and centrifugation. Appl Environ Microbiol.

[10] Shih HY, Lin YE (2006). Caution on interpretation of legionella results obtained using real-time PCR for environmental water samples. Appl Environ Microbiol.

[11] Mercante JW, Winchell JM (2015). Current and emerging Legionella diagnostics for laboratory and outbreak investigations. Clin Microbiol Rev.

[12] Diederen BM, Kluytmans JA, Vandenbroucke-Grauls CM, Peeters MF (2008). Utility of real-time PCR for diagnosis of Legionnaires' disease in routine clinical practice. J Clin Microbiol.

[13] Krøjgaard LH, Krogfelt KA, Albrechtsen HJ, Uldum SA (2011). Detection of Legionella by quantitative-polymerase chain reaction (qPCR) for monitoring and risk assessment. BMC Microbiol.

[14] Whiley H, Taylor M (2016). Legionella detection by culture and qPCR: comparing apples and oranges. Crit Rev Microbiol.

[15] Wang Y, Wang Y, Ma AJ, Li DX, Luo LJ, Liu DX (2015). Rapid and sensitive isothermal detection of nucleic-acid sequence by multiple cross displacement amplification. Sci Rep.

[16] Wang Y, Wang Y, Zhang L, Liu D, Luo L, Li H (2016). Multiplex, rapid, and sensitive isothermal detection of nucleic-acid sequence by endonuclease restriction-mediated real-time multiple cross displacement amplification. Front Microbiol.

[17] Li S, Liu C, Liu Y, Ma Q, Wang Y, Wang Y (2019). Development of a multiple cross displacement amplification combined with nanoparticles-based biosensor assay to detect Neisseria meningitidis. Infect Drug Resist.

[18] Li S, Jiang W, Huang J, Liu Y, Ren L, Zhuang L, et al. Highly sensitive and specific diagnosis of coronavirus disease 19 (COVID-19) by reverse transcription multiple cross displacement amplification-labelled nanoparticles biosensor. Eur Respir J. 2020;56(6):2002060. 10.1183/13993003.02060-2020 PMC7453731 32859676

[19] Jiang L, Li X, Gu R, Mu D (2020). Nanoparticles-Based Biosensor Coupled with Multiplex Loop-Mediated Isothermal Amplification for Detection of Staphylococcus aureus and Identification of Methicillin-Resistant S. aureus. Infect Drug Resist.

[20] Zhu X, Wang X, Han L, Chen T, Wang L, Li H (2020). Multiplex reverse transcription loop-mediated isothermal amplification combined with nanoparticle-based lateral flow biosensor for the diagnosis of COVID-19. Biosens Bioelectron.

[21] Yong SF, Tan SH, Wee J, Tee JJ, Sansom FM, Newton HJ (2010). Molecular detection of Legionella: moving on from mip. Front Microbiol.

[22] Quesada-González D, Merkoçi A (2015). Nanoparticle-based lateral flow biosensors. Biosens Bioelectron.

[23] Yrad FM, Castañares JM, Alocilja EC. Visual Detection of Dengue-1 RNA Using Gold Nanoparticle-Based Lateral Flow Biosensor. Diagnostics (Basel, Switzerland). 2019;9(3):74. 10.3390/diagnostics9030074 PMC6787709 31336721

[24] Jiao WW, Wang Y, Wang GR, Wang YC, Xiao J, Sun L (2019). Development and clinical validation of multiple cross displacement amplification combined with nanoparticles-based biosensor for detection of mycobacterium tuberculosis: preliminary results. Front Microbiol.

[25] Tabatabaei M, Hemati Z, Moezzi MO, Azimzadeh N (2016). Isolation and identification of Legionella spp. from different aquatic sources in south-west of Iran by molecular &culture methods. Mol Biol Res Commun.

[26] Moosavian M, Seyed-Mohammadi S, Saki M, Shahi F, Khoshkholgh Sima M, Afshar D (2019). Loop-mediated isothermal amplification for detection of Legionella pneumophila in respiratory specimens of hospitalized patients in Ahvaz, Southwest Iran. Infect Drug Resist.

