# Supplementary material for: Multiple Cross Displacement Amplification Coupled with Lateral Flow Biosensor (MCDA-LFB) for rapid detection of Legionella pneumophila
Source: BMC Microbiol. 2022 Jan 8;22:20. doi: 10.1186/s12866-021-02363-3 (PMC8742375; doi:10.1186/s12866-021-02363-3)
Supplement: Supplementary file 1 — Additional file 1. [file 12866_2021_2363_MOESM1_ESM.docx]

| **Additional table 1：**Information on 23 strains of *L. pneumophila* | | |  |
| --- | --- | --- | --- |
| **Serial number** | **Environment source** | **L. pneumophila serogroup (Sg)** | **ST type** |
| 1 | Hospital pond water | Sg1 | ST1 |
| 2 | Hospital pond water | Sg1 | ST1 |
| 3 | Sputum specimen | Sg7 | ST35 |
| 4 | Bronchoscopy alveolar lavage fluid | Sg1 | ST1 |
| 5 | Sputum specimen | Sg6 | ST222 |
| 6 | Sputum specimen | Sg1 | ST1 |
| 7 | Hospital pond water | Sg1 | ST1 |
| 8 | Bronchoscopy alveolar lavage fluid | Sg3 | ST328 |
| 9 | Hospital pond water | Sg1 | ST328 |
| 10 | Bronchoscopy alveolar lavage fluid | Sg1 | ST1 |
| 11 | Sputum specimen | Sg1 | ST1 |
| 12 | Bronchoscopy alveolar lavage fluid | Sg1 | ST1 |
| 13 | Bronchoscopy alveolar lavage fluid | Sg1 | ST1 |
| 14 | Sputum specimen | Sg3 | ST36 |
| 15 | Bronchoscopy alveolar lavage fluid | Sg1 | ST1 |
| 16 | Hospital pond water | Sg1 | ST37 |
| 17 | Sputum specimen | Sg1 | ST1 |
| 18 | Bronchoscopy alveolar lavage fluid | Sg1 | ST1 |
| 19 | Bronchoscopy alveolar lavage fluid | Sg1 | ST1 |
| 20 | Sputum specimen | Sg1 | ST1 |
| 21 | Hospital pond water | Sg1 | ST1 |
| 22 | Sputum specimen | Sg1 | ST1 |
| 23 | Sputum specimen | Sg1 | ST1 |

| **Additional table 2:** Information on sputum, alveolar lavage fluid and water specimens |
| --- |

| Serial number | Sample source | Collection date | Severity of pneumonia | PCR | Culture | MCDA-LFB |
| --- | --- | --- | --- | --- | --- | --- |
| 1 | Sputum | 2019.10.14 | severe | Negative | Negative | Negative |
| 2 | Sputum | 2019.10.15 | severe | Negative | Negative | Negative |
| 3 | Sputum | 2019.10.16 | mild | Negative | Negative | Negative |
| 4 | Sputum | 2019.10.18 | severe | Negative | Negative | Negative |
| 5 | Sputum | 2019.10.22 | severe | Negative | Negative | Negative |
| 6 | Sputum | 2019.10.22 | severe | Negative | Negative | Negative |
| 7 | Sputum | 2019.10.25 | severe | Negative | Negative | Negative |
| 8 | Sputum | 2019.10.26 | severe | Negative | Negative | Negative |
| 9 | Sputum | 2019.11.04 | severe | Negative | Negative | Negative |
| 10 | Sputum | 2019.11.06 | mild | Positive | Positive | Positive |
| 11 | Sputum | 2019.11.06 | severe | Negative | Negative | Negative |
| 12 | Sputum | 2019.11.08 | severe | Negative | Negative | Negative |
| 13 | Sputum | 2019.11.12 | mild | Negative | Negative | Negative |
| 14 | Sputum | 2019.11.13 | severe | Negative | Negative | Negative |
| 15 | Sputum | 2019.11.15 | severe | Negative | Negative | Negative |
| 16 | Sputum | 2019.11.15 | severe | Negative | Negative | Negative |
| 17 | Sputum | 2019.11.17 | severe | Positive | Positive | Positive |
| 18 | Sputum | 2019.11.21 | severe | Negative | Negative | Negative |
| 19 | Sputum | 2019.11.21 | severe | Negative | Negative | Negative |
| 20 | Sputum | 2019.11.22 | severe | Negative | Negative | Negative |
| 21 | Sputum | 2019.11.22 | severe | Negative | Negative | Negative |
| 22 | Sputum | 2019.11.22 | severe | Negative | Negative | Negative |
| 23 | Sputum | 2019.11.22 | severe | Negative | Negative | Negative |
| 24 | Sputum | 2019.11.25 | severe | Negative | Negative | Negative |
| 25 | Sputum | 2019.11.25 | severe | Negative | Negative | Negative |
| 26 | Sputum | 2019.11.26 | severe | Positive | Positive | Positive |
| 27 | Sputum | 2019.11.27 | severe | Negative | Negative | Negative |
| 28 | Sputum | 2019.11.29 | severe | Negative | Negative | Negative |
| 29 | Sputum | 2019.11.29 | severe | Negative | Negative | Negative |
| 30 | Sputum | 2019.12.02 | severe | Negative | Negative | Negative |
| 31 | Sputum | 2019.12.02 | mild | Negative | Negative | Negative |
| 32 | Sputum | 2019.12.02 | severe | Negative | Negative | Negative |
| 33 | Sputum | 2019.12.05 | severe | Negative | Negative | Negative |
| 34 | Sputum | 2019.12.07 | severe | Negative | Negative | Negative |
| 35 | Sputum | 2019.12.08 | severe | Negative | Negative | Negative |
| 36 | Sputum | 2019.12.11 | severe | Negative | Negative | Negative |
| 37 | Sputum | 2019.12.11 | mild | Negative | Negative | Negative |
| 38 | Sputum | 2019.12.13 | severe | Negative | Negative | Negative |
| 39 | Sputum | 2019.12.14 | severe | Negative | Negative | Negative |
| 40 | Sputum | 2019.12.14 | severe | Negative | Positive | Positive |
| 41 | Sputum | 2019.12.16 | severe | Negative | Negative | Negative |
| 42 | Sputum | 2019.12.17 | severe | Negative | Negative | Negative |
| 43 | Sputum | 2019.12.17 | mild | Negative | Negative | Negative |
| 44 | Sputum | 2019.12.19 | severe | Negative | Negative | Negative |
| 45 | Sputum | 2019.12.19 | severe | Negative | Negative | Negative |
| 46 | Sputum | 2019.12.20 | severe | Negative | Negative | Negative |
| 47 | Sputum | 2019.12.24 | severe | Negative | Negative | Negative |
| 48 | Sputum | 2019.12.27 | severe | Negative | Negative | Negative |
| 49 | Sputum | 2019.12.27 | mild | Negative | Negative | Negative |
| 50 | Sputum | 2019.12.30 | mild | Negative | Negative | Negative |
| 51 | Sputum | 2019.12.30 | severe | Negative | Negative | Negative |
| 52 | Sputum | 2019.12.31 | severe | Negative | Negative | Negative |
| 53 | Sputum | 2020.01.03 | severe | Negative | Negative | Negative |
| 54 | Sputum | 2020.01.03 | severe | Negative | Negative | Negative |
| 55 | Sputum | 2020.01.06 | severe | Negative | Negative | Negative |
| 56 | Sputum | 2020.01.08 | severe | Negative | Negative | Negative |
| 57 | Sputum | 2020.01.08 | mild | Negative | Negative | Negative |
| 58 | Sputum | 2020.01.08 | severe | Negative | Negative | Negative |
| 59 | Sputum | 2020.01.10 | severe | Negative | Negative | Negative |
| 60 | Sputum | 2020.01.10 | severe | Negative | Negative | Negative |
| 61 | Sputum | 2020.01.11 | severe | Negative | Negative | Negative |
| 62 | Sputum | 2020.01.12 | severe | Negative | Negative | Negative |
| 63 | Sputum | 2020.01.15 | severe | Negative | Negative | Negative |
| 64 | Sputum | 2020.01.16 | severe | Negative | Negative | Negative |
| 65 | Sputum | 2020.01.20 | severe | Negative | Negative | Negative |
| 66 | Sputum | 2020.01.20 | severe | Positive | Positive | Positive |
| 67 | Sputum | 2020.01.22 | severe | Negative | Negative | Negative |
| 68 | Sputum | 2020.01.23 | mild | Negative | Negative | Negative |
| 69 | Sputum | 2020.02.25 | severe | Negative | Negative | Negative |
| 70 | Sputum | 2020.02.27 | severe | Negative | Negative | Negative |
| 71 | Sputum | 2020.02.28 | severe | Negative | Negative | Negative |
| 72 | Sputum | 2020.02.28 | severe | Negative | Negative | Negative |
| 73 | Sputum | 2020.03.03 | severe | Negative | Negative | Negative |
| 74 | Sputum | 2020.03.09 | severe | Negative | Negative | Negative |
| 75 | Sputum | 2020.03.12 | mild | Negative | Negative | Negative |
| 76 | Sputum | 2020.03.17 | severe | Negative | Negative | Negative |
| 77 | Sputum | 2020.03.19 | severe | Negative | Negative | Negative |
| 78 | Sputum | 2020.03.22 | severe | Negative | Negative | Negative |
| 79 | bronchoalveolar lavage fluid | 2020.06.24 | severe | Negative | Negative | Negative |
| 80 | bronchoalveolar lavage fluid | 2020.07.07 | severe | Negative | Negative | Negative |
| 81 | bronchoalveolar lavage fluid | 2020.07.12 | severe | Negative | Negative | Negative |
| 82 | bronchoalveolar lavage fluid | 2020.07.21 | severe | Negative | Negative | Negative |
| 83 | bronchoalveolar lavage fluid | 2020.07.24 | severe | Negative | Negative | Negative |
| 84 | water | 2020.08.17 | NA | Negative | Negative | Negative |
| 85 | Water | 2020.08.20 | NA | Negative | Negative | Negative |
| 86 | Water | 2020.08.24 | NA | Negative | Negative | Negative |
| 87 | Water | 2021.05.06 | NA | Negative | Negative | Negative |
| 88 | water | 2021.05.20 | NA | Negative | Negative | Negative |
